# Women’s Empowerment and Children’s Complete Vaccination in the Democratic Republic of the Congo: A Cross-Sectional Analysis

**DOI:** 10.3390/vaccines9101117

**Published:** 2021-10-01

**Authors:** Xinran Lu, Chuchu Fu, Qianyun Wang, Qiwei He, Jiayi Hee, Rie Takesue, Kun Tang

**Affiliations:** 1 Vanke School of Public Health, Tsinghua University, Beijing 100084, China; luxr@pku.edu.cn (X.L.); heqiwei@mail.tsinghua.edu.cn (Q.H.); 2 School of Public Health, Peking University, Beijing 100083, China; 3 College of Humanities and Development Studies, China Agricultural University, Beijing 100193, China; fuchuchu@cau.edu.cn; 4 Institute of Population Research, Peking University, Beijing 100871, China; wangqianyun@stu.pku.edu.cn; 5 School of Public Health, The University of Queensland, Herston, QLD 4006, Australia; j.hee@uqconnect.edu.au; 6 UNICEF Headquarters, New York, NY 10017, USA; rtakesue@unicef.org

**Keywords:** women’s empowerment, complete vaccination, the Democratic Republic of the Congo, intrinsic agency, enabling resources

## Abstract

(1) Background: The empowerment of women contributes to better child health and wellness. This study aimed to examine the association between women’s empowerment and complete vaccination of children, as recommended in the National Expanded Program on Immunization (EPI) in the Democratic Republic of the Congo (DRC). (2) Methods: In this cross-sectional study, a principal component analysis (PCA) was conducted on data from the Multiple-Indicator Cluster Survey 6 (MICS-6) to determine the dimensions of women’s empowerment. Logistic regression analysis was used to assess the association between women’s empowerment and complete vaccination of children stratified by household wealth. In total, 3524 women with children aged 12–23 months were included in the study. (3) Results: Women’s empowerment was defined by three dimensions, namely intrinsic agency, enabling resources, and social independence. Children of women with high levels of empowerment had higher odds of complete vaccination, with values of 1.63 (*p* = 0.002) and 1.59 (*p* = 0.012) for intrinsic agency and enabling resources of the empowerment, respectively, compared to the children of women with low levels of empowerment; however, social independence failed to be associated with the vaccination status of children. After stratification by household wealth, the OR of complete vaccination was higher in women from middle-income households with high levels of intrinsic agency (OR: 2.35, *p* = 0.021) compared to women from poor households with high levels of intrinsic agency (OR: 1.92, *p* = 0.004). (4) Conclusions: Higher levels of women’s empowerment, especially intrinsic agency and enabling resources, were associated with complete vaccination in children in the DRC. Household wealth status influenced the associations. The empowerment of women is crucial in promoting the complete vaccination of children and providing equal access to vaccines.

## 1. Introduction

The complete vaccination of children remains a global health issue. According to the World Health Organization (WHO), global vaccination coverage has plateaued over the past few years [1]. In 2019, 60% of countries, including most African countries, failed to achieve the WHO national vaccination coverage goal of 90% for all scheduled vaccines [2]. Meanwhile, the burden of vaccine-preventable disease (VPD) remains high in low- and middle-income countries [3]. The WHO reported that among 19.7 million infants who did not receive any diphtheria–tetanus–pertussis (DTP) vaccination or who were only partially vaccinated, more than 60% these of children were concentrated in 10 countries. This included the Democratic Republic of the Congo (DRC), with a DTP coverage of 63% in 2010 [1,4]. The DRC experienced a resurgence of measles in 2010 [5,6], an outbreak of yellow fever with vaccine shortage in 2016 [7,8], and multiple independent events of the emergence of circulating vaccine-derived poliovirus strains from 2004 to 2018 [9,10,11,12]. Meanwhile, the full vaccination coverage of children aged 12–23 months was about 45.3% in 2013–2014 [13] and measles vaccine effectiveness among children aged 12–59 months was 80% in 2010–2012, which was lower than expected [14]. The evidence underscores the necessity for the enhancement of vaccine uptake and the improvement of vaccine delivery strategies in the DRC.

The empowerment of women contributes to better health in children, as women often take on the role of the primary caregivers in the family and are more willing to spend on their children compared to men [15,16]. As gender-based approaches are increasingly adopted to scrutinize health inequities, it has been revealed that the empowerment of mothers tends to result in better maternal and child health outcomes, such as higher children immunization coverage, increased maternal health service utilization, and improvement in the nutritional status of children [17,18,19,20]. Researchers have examined the association between the empowerment of mothers and vaccination of children in low- and middle-income countries to explore empowerment pathways of the “maternal resource-child vaccination” relationship [19,21]. In some studies, women’s agency was found to be positively associated with the complete vaccination of children in African countries [22,23,24].

Although researchers have tried to scientifically describe and measure the empowerment of women, no consensus on the best available tool to measure the empowerment of women has been reached. Empowerment is commonly perceived as enhancing or reacquiring the ability to make choices and take control over their lives, which is the process as well as the outcome from both collective and individual dimensions [5]. For women, empowerment can be hypothesized as to how to exercise power within the gender system [5,20]. On the individual level, Kabeer (1999) stressed that women’s empowerment incorporated acquiring enabling resources (including material, human, and social resources), exercise agency (including processes of decision making with the capacity of planning, manipulation, negotiation, and mobilizing available resources, etc.), and attainment of life achievements (such as well-being outcomes), which are commonly adopted afterward [24,25,26]. Highlighted as the key component of empowerment, self-agency was defined as the ability to define one’s goals and act upon them [25]. Thorpe et al. (2016) focused on decision-making and freedom of movement to measure women’s agency [24]. Miedema et al. (2018) operationalized intrinsic agency as attitudes towards wife-beating and sexual activity [26]; however, there remain inconsistencies in the definition and operationalization of the concept of women’s agency.

Regarding the widely used indicators of gender development in the extant literature, these are mainly operationalized from dimensions of socioeconomic resources, maternal health, self-agency, and familial relationships. Indicators of socioeconomic empowerment include women’s literacy and education, income, employment, and personal ownership of property [27,28,29,30,31]. Maternal-health-related indicators of empowerment include marital status; access to reproductive health services; women’s knowledge about diseases; and ability to decide when to get married, when to have children, and how many children to have [16,21,32,33,34,35]. Indicators of women’s agency within the gender system include their involvement in household decisions, access to information, ability to exercise mobility choices, and attitudes towards physical and sexual violence [36,37,38,39,40,41]. Indicators of relationship consist of differences in age and education between couples and the woman’s age at major life events, including first cohabitation, first marriage, first sexual experience, and first birth [21,23,27,33]. As such, studies concerning the multidimensional measurement of women’s empowerment may help to determine specified factors that affect the maternal and child health outcomes under the gender framework.

Of all the factors associated with satisfactory vaccination uptake, maternal empowerment may be one of the major determinants [39]. Relevant studies focused on South Asia (such as India, Pakistan, and Nepal) [21,39,40,41,42], West Africa (such as Nigeria and Ghana) [30,43], and East Africa (such as Ethiopia and Kenya) [23,29,31,38], but few studies have focused on Central Africa. Considering the prevalence of sexual violence in the DRC [44,45], increased awareness of women’s empowerment is necessary. Therefore, using the United Nations Children’s Fund (UNICEF) Multiple-Indicator Cluster Survey 6 (MICS-6) conducted in the DRC, the present study seeks to contribute to the women empowerment–child vaccination literature by measuring women’s empowerment in the DRC using principal component analysis (PCA) to examine the associations among several dimensions of women’s empowerment and the complete vaccination of children. As one of the first studies to shed light on the association between maternal empowerment and vaccination of children using the MICS data, and being the first study to investigate this association in the DRC, this study seeks to help promote the implementation of relevant interventions and policies, which are also embodied in the Sustainable Development Goals (SDGs) for world development.

## 2. Materials and Methods

### 2.1. Study Participants

**Figure 1:**
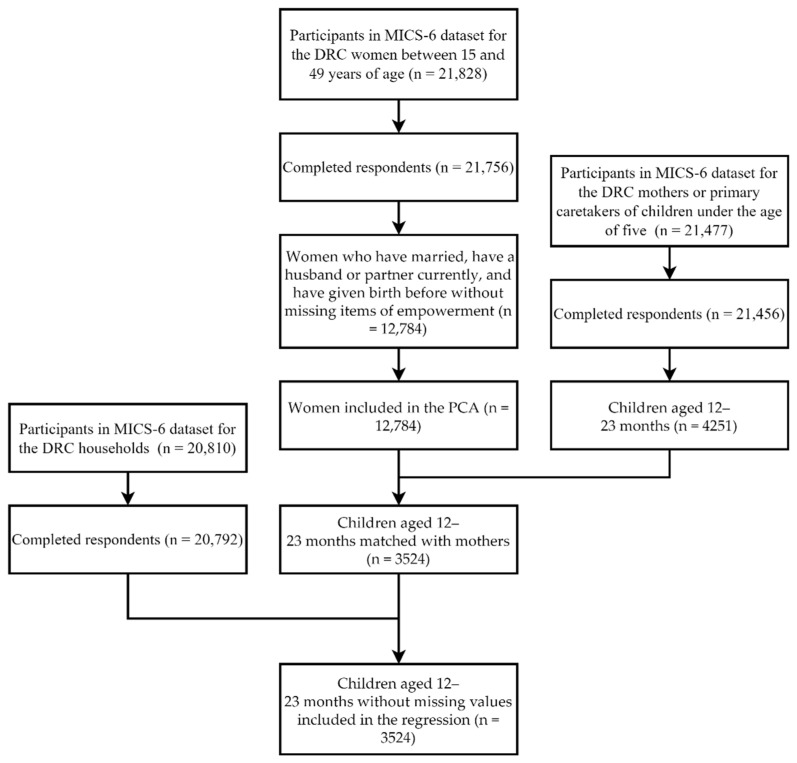
Flowchart of the selection process.

For this cross-sectional study, we extracted data from the MICS-6 in the DRC, conducted in 2017 and 2018 by the National Statistics Institute of the DRC Ministry of Planning, in collaboration with UNICEF, as part of the MICS global survey program. The MICS developed by UNICEF aims to collect internationally comparable data on a wide range of variables related to the status of children and women. In short, the MICS-6 in the DRC used a two-stage stratified cluster sampling design to select 721 clusters and 21,630 households [46]. Three questionnaires were used for our analysis, one for women between 15 and 49 years of age, one for mothers or primary caretakers of children under the age of five, and the other for households. The selection process is presented in Figure 1. The inclusion criteria for PCA were that the women must either be married or currently have a partner and have given birth before, without missing items of empowerment. In total, 12,784 responses with full data were retained for the measurement of women’s empowerment in the DRC. A child was matched to a woman if the ID number of the child’s caretaker was the same as the woman’s ID number. In total, 3524 children aged 12–23 months matched with mothers without missing values were included in the regression analysis of this study. The MICS data are publicly available on the UNICEF website [47]. This study has been authorized by UNICEF and is exempted from further ethical review.

### 2.2. Framework

Our analysis was built on Kabeer’s framework of empowerment of women [25], incorporating resources, agency, and achievement. The resources include financial support, as well as the more indispensable resources, namely social resources, which serve as a prerequisite to exert power. In terms of the second dimension, agency represents a more intangible ability to act in a specific condition, which can be operationalized as an attitude of revolt and resistance, or to be more observable, the act of decision-making. While achievement was the health outcome of childhood vaccination in the present study, the domain was excluded in the context of maternal empowerment. Kabeer concentrated on the process of exercising choice, yet failed to accord with the context of the decision for children’s health access. As such, we referred to the survey-based Women’s Empowerment (SWPER) index [27] and set indicators of social independence in our framework. As family decisions often involve both parents, as well as other family members, the family background of the women should be taken into consideration. In general, our framework consisted of agency, resources, and setting.

### 2.3. Measures

Women’s empowerment was measured using 13 questions extracted from the questionnaires for women between 15 and 49 years of age in MICS-6 from the DRC to embody the three-dimensional framework. These 13 items were identified based on Kabeer’s framework [25], Miedema’s model in East Africa [26], and the SWPER index [27], including women’s attitudes toward domestic violence, highest education level, health insurance coverage, use of mass media and information and communications technology, the age difference between women and their partners, and ages at first marriage and first birthing. The details and definitions of each item are shown in Table S1 in the Supplementary Materials.

The National Expanded Program on Immunization (EPI) in the DRC recommends that all children are vaccinated for 1 dose of Bacilli Calmette–Guerin (BCG) vaccine; 3 doses of polio vaccines (excluding 1 dose of polio vaccine at birth); 3 doses of the pentavalent vaccines containing antigens against diphtheria, tetanus, pertussis, hepatitis B, and Hemophilus influenza type b (Hib); 3 doses of pneumococcal conjugate vaccines (PCV); 1 dose of measles vaccine; and 1 dose yellow fever vaccine before their first birthday [46]. “Complete vaccination”, which is the primary outcome, was defined as children aged 12–23 months who had received all vaccines in the EPI, while “incomplete vaccination” was defined as children who had missed one or more doses of recommended vaccines. The related questions are provided in the immunization sections from the questionnaires for mothers or primary caretakers of children under the age of five. The vaccination record was acquired via the vaccination card; however, if the vaccination card was unavailable when the survey was conducted, details of the vaccination were based on the mother’s recollection.

In addition, the age of the women, gender of the children, and household characteristics were included to assess the association with children’s vaccination. Household characteristics included urban or rural residence, family wealth index, the number of household members, children under 5 years of age, and province. The family wealth index in MICS was presented as five quintiles and was recoded into poor (the first two quintiles), middle (the third quintile), and rich (the last two quintiles). The number of household members was categorized as 1–5, 6–10, and ≥10. The number of children aged under 5 years old in the household was classified into 1, 2, and ≥3.

### 2.4. Statistical Analysis

The principal component analysis (PCA) was conducted to determine the dimensions of women’s empowerment. PCA is a frequently used method to reduce the dimensionality of large datasets, while preserving most of the variability and statistical information for all variables [25]. The varimax orthogonal rotation was applied to retain the uncorrelated factors [27]. The factor scores of each domain were calculated and evenly distributed across three levels (low, middle, and high) for further analyses. Descriptive analysis was used to describe the variables of women’s empowerment and the characteristics of women, children, and their households for total participants and different vaccination statuses of the children. Continuous variables were described using means and standard deviations and categorical variables were summarized as proportions. To test the statistical differences between different groups, Student’s t-tests were used for continuous variables and Wald Chi-square tests were used for categorical variables. To investigate the associations between women’s empowerment and children’s vaccination, logistic regressions controlled for women’s age, children’s gender and household factors were conducted. Due to the influence of the wealth level on children’s vaccination status [21,31,48,49] and women’s empowerment [50], we assessed the heterogeneity of different family wealth indexes using logistic regressions within each wealth status to investigate the role of wealth between empowerment and vaccination. We reported the odds ratios (ORs), 95% confidence intervals (CIs), and *p*-values of all regressions. The descriptive analysis and regression analyses were weighted according to the sampling weights provided by the MICS. All statistical analyses were performed using Stata version 16.0 (College Station, TX 77845, USA).

## 3. Results

### 3.1. Dimensions of Women’s Empowerment

Table 1 shows the factor loadings for the 13 items in MICS after varimax orthogonal rotation. The factor loadings measured the variance of each item, which can be explained by the factors and which indicated the positive or negative relationships between items and factors. Items with factor loadings higher than 0.3 were identified to be attributed to the factor. Considering Kaiser’s criterion [51], namely that factors with eigenvalues ≥ 1 should be kept, as well as the result of the scree plot with the turning point at the fourth dot (Figure S1), four factors should be extracted; however, as the fourth factor was dominated by items similar to the first three factors, the eigenvalue of the fourth factor was very close to 1 (eigenvalue: 1.039). As such, the first three factors were retained for further analysis. The appendix (Table S2) provides the PCA rotated factor loadings for four factors. The first three factors accounted for 23.9%, 15.6%, and 12.7% of the variance, adding up to 52.6% (Table S3).

We defined the first three factors in order of eigenvalue magnitude as the intrinsic agency, enabling resources, and social independence, corresponding to the framework of agency, resources, and setting and specifically presenting the content of every dimension. Intrinsic agency was defined according to 5 questions regarding the attitudes of women towards their husband’s violence, whereby “beating not justified if”: (1) “wife goes out without telling husband”; (2) “wife neglects the children”; (3) “wife argues with husband”; (4) “wife refuses to have sex with husband”; (5) “wife burns the food”. Enabling resources was defined according to women’s highest education level, health insurance coverage, possession of a mobile phone, having used the internet, and having used a computer. Social independence was defined according to “the age difference between women and their husband”, “women’s age at first marriage”, and “women’s age at first birthing”.

A series of tests were performed to assess the fit of the PCA. The Kaiser–Meyer–Olkin (KMO) measure indicated an overall KMO testing score of 0.74, while Bartlett’s test of sphericity was significant for χ^2^ = 41991.40, *p* < 0.001, indicating a sufficiently strong correlation among the items, which proved the suitability of PCA for our study [52].

### 3.2. Characteristics of Study Participants

The characteristics of the women, children, and households according to the vaccination status of the children are presented in Table S4. The mean age of women was 29.6 years, ranging 15–49 years. Half of the children were male (50.1%), 60.6% of women reported living in a rural area, 45.3% of the participants were from households with low household wealth, and 35.1% of the participants were from households with high household wealth.

The characteristics of women’s empowerment according to the vaccination status of their children are presented in Table 2. According to the EPI in the DRC, 36.6% of children in this sample had been completely vaccinated. The coverage level for each vaccine in the EPI is presented in the appendix (Table S5), ranging from 45.8% (PCV) to 73.2% (BCG). Among the different situations of violence from women’s husbands, more than half of the women believed that domestic violence towards them was not justified. This ranged from 53.8% when she argued with her husband to 75.4% when she burned the food. About half of the women (48.2%) had educational levels above primary school. On average, the women were 6.7 years younger than their husbands, were married at 19.0 years of age, and delivered their first child at 20.7 years of age. Only a small percentage of women reported that they were covered by health insurance (3.8%), had used the Internet (4.4%), or had used a computer (5.2%). All variables regarding empowerment significantly correlated to vaccination status, except for the age difference between a woman and her husband, which was attributed to social independence.

### 3.3. Association between Women’s Empowerment and Children’s Vaccination Status

Table 3 shows the association between women’s empowerment and children’s complete vaccination. After adjusting for all possible variables, intrinsic agency and enabling resources of the women were observed to be significantly associated with complete vaccination; however, social independence failed to be significantly associated with the outcome variable. The odds of complete vaccination in children from women with high levels of intrinsic agency or enabling resources were 1.63 (95%CI: 1.19–2.24, *p* = 0.002) or 1.59 (95%CI: 1.11–2.28, *p* = 0.012), respectively, compared to children from women with low levels of empowerment. The odds of complete vaccination were lower in urban residences (OR: 0.66, 95%CI: 0.46–0.95, *p* = 0.027) compared with rural residences. In children of women from middle or rich households, the odds of complete vaccination of children were 1.50 (95%CI: 1.07–2.09, *p* = 0.018) and 3.06 (95%CI: 1.96–4.78, *p* < 0.001), respectively, compared to children of women from poor households. It was also observed that the odds of complete vaccination were lower in households with two or three and more children under the age of 5 (OR: 0.71, 95%CI: 0.53–0.94, *p* = 0.015; OR: 0.66, 95%CI: 0.45–0.98, *p* = 0.039) respectively, compared with households with one child under the age of 5. Women from provinces except for Kwilu, Mongala, Maniema, and Lualaba were more likely to have their children be completely vaccinated as compared to Sankuru, the province with the lowest complete vaccination coverage in the DRC. The odds of complete vaccination of children ranged from 4.53 in Maindombe (CI: 1.08–19.01, *p* = 0.039) to 74.83 in Nord Kivu (CI: 18.43–303.75, *p* < 0.001) compared to those in Sankuru.

Findings from the stratified analysis by household wealth status revealed that children’s vaccination had a significant association with women’s intrinsic agency in the poor and middle wealth categories, women’s enabling resources in the middle wealth and rich categories, and women’s social independence in the rich category (Table 4). For children of women with low household wealth and middle or high levels of intrinsic agency, the odds of complete vaccination were 1.72 (95%CI: 1.09–2.70, *p* = 0.019) and 1.92 (95%CI: 1.23–2.98, *p* = 0.004), respectively, compared to children of women with low household wealth and low levels of intrinsic agency. Similarly, among children of women with middle household wealth and high levels of intrinsic agency, the odds of complete vaccination were 2.35 (CI: 1.14–4.85, *p* = 0.021) compared to children of women with middle household wealth and low levels of intrinsic agency. The odds of complete vaccination were higher in women from the middle-income households with high levels of intrinsic agency compared to women from poor households with high levels of intrinsic agency. Among children of women with middle household wealth and middle levels of enabling resources, the odds of complete vaccination were 2.19 (95%CI: 1.10–4.36, *p* = 0.026) compared to children of women with middle household wealth and low levels of enabling resources. In children of women from a rich household with a high level of enabling resources, the odds of complete vaccination were 2.62 (95%CI: 1.01–6.78, *p* = 0.047) compared to children of women from a rich household with a low level of enabling resources. Furthermore, among children of women with high household wealth and middle levels of social independence, the odds of complete vaccination were 0.47 (95%CI: 0.26–0.83, *p* = 0.010) compared to children of women with high household wealth and low levels of social independence.

## 4. Discussion

This study measured the empowerment of women as their intrinsic agency, enabling resources, and social independence using PCA. It also investigated the associations among different dimensions of women’s empowerment and children’s vaccination in the DRC. The intrinsic agency and enabling resources of women showed a strong positive effect on children’s vaccination status, while the social independence failed to influence children’s vaccination status. Children of women who strongly opposed domestic violence or possessed more social resources were more likely to be completely vaccinated. Apart from this, the results showed that complete vaccination of children was associated with higher household income, rural residency, and fewer children aged under 5 in the family. After stratification of household wealth, the effect of women’s empowerment on their children’s vaccination status was greatly associated with familial economic status. A high level of intrinsic agency was found to be associated with complete vaccination in the women from a poor household, whereas enabling resources showed a similar effect in the women from a rich household.

There is no consensus on the measurement of the term women’s empowerment. The goal of the present study was to measure this notion using existing databases to explain the maternal influence on their children’s vaccination. We interpreted the notion as the intrinsic agency, enabling resources, and social independence. Agency contributed the highest proportion among the three factors to women’s empowerment, indicating its salient role of linkage between resources and outcome. Disapproval of domestic violence represented the intrinsic agency, while the enabling resources included education level and other power to handle resources. Social independence represented the relationship between the women and their husbands using age-related items, as well as ages at pivotal life events. Women’s acquiescence to marital violence undermines their power towards husbands and suggests a subordinate status in their families, meaning this is an appropriate indicator to represent intrinsic agency. Furthermore, participation in reproductive health care, household decisions, and public affairs are regarded as the other measures in terms of intrinsic agency, while the domain of enabling resources also includes women’s income and employment [15,16,26,32,53]; however, as these items were beyond the scope of our study and unavailable in the database, further studies utilizing more appropriate survey designs to measure women’s empowerment are imperative.

The pattern of the association between women’s empowerment and children’s complete vaccination was similar to previous studies [13,21,24,32,35,39,54,55,56,57,58,59,60]. Women’s disapproval attitude towards violence increased access to health services, which resulted in better health statuses in children. This included the reduction of child stunting and malnutrition [55,59,60]. It could be argued that the acceptance of violence portrays submissiveness of the wife and the dominion of the husband, including in allocating income, deciding expenditures, and determining access to health services for their children. This confirmed that women who possess more autonomous decision-making power were more likely to vaccinate their children [24,54]. Our study also demonstrated strong positive effects of maternal material and social resources on their children. This suggested that the possession of more resources, including women’s education, health insurance, communication techniques, and usage of mass media, results in greater possibility of children’s complete vaccination, corresponding to the findings of other studies conducted in the DRC [13,56] and other countries [21,35,39,58]. Maternal education has repeatedly been proven to be a vital determinant of children’s vaccination status [13,21,35,39,54,56,58]. Additionally, access to information and media may also result in better health status of children, as measured by their vaccination status [41] and history of pneumonia and anemia [57]. The educational attainment and information from media may enhance the access and absorption of knowledge and literacy, as well as communication with health workers, leading to a better comprehension of EPI and complete vaccination. On account of the higher proportion of catastrophic expenditure in the DRC, it is suggested that health insurance should be ensured to alleviate familial economic burden [61]. Health insurance was found to improve children’s nutritional status [20] and complete the basic immunization for children [43]. These social resources serve as indirect indicators of empowerment and provide women with opportunities to develop their capabilities and power needed for agency, improving their children’s health access.

Nevertheless, social independence revealed no influence on the vaccination status of children in the DRC, corresponding to studies from Nepal and Kenya; that is, maternal early marriage, maternal age at first birth, and the gap in age between spouses were not associated with child vaccination [21,23]. On the other hand, a regional study in sub-Saharan Africa and southwest Asia showed that children would more likely receive fundamental vaccinations if their mothers married at the age of 15–17 instead of 10–14 [33]. The potential reason for the inconsistency was that the regional study concealed the specific socioeconomic situations of every country and using the categorical variables of age, while the present study, similar to the two national research studies, focused on the context and used continuous variables of maternal age at pivotal life events. Furthermore, social independence in the present study included three items in terms of the women’s social interactions with their families, especially with their husbands. The insignificance of this to children’s vaccination status may have been due to the generally low levels of different aspects of empowerment in the DRC, underscoring the need to focus on more items related to empowerment to detect its influence on health outcomes.

Wealth not only has a strong influence on the vaccination status [21,48,49] but also influences the relationship between women’s empowerment and health outcomes [23,53,62]. In the wealth-stratified analysis in the present study, the positive effect of women’s empowerment, especially intrinsic agency and enabling resources, on the vaccination of children was strengthened as the household wealth status increased. Similarly, a study from Kenya reported a threshold effect of household wealth, indicating that once a certain wealth level is reached, a high level of women’s empowerment would be able to promote the vaccination of children, owing to the availability of financial resources [23]. Research on the connection between empowerment of women and their own nutritional status [53] or their children’s [62] also proved the potential threshold effect. It was shown by Kabeer (1999) [25] that wealth could be regarded as a fundamental component or prerequisite for empowerment, so that the more financial or social assets a woman owns, the more resources and knowledge they possess to enable vaccination and other health outcomes. The results also illustrated that intrinsic agency has a significant effect on families with low household wealth, while resources have a significant effect on families with high household wealth. Two studies utilizing the Demographic and Health Surveys (DHS) data in different countries found that improving intrinsic agency for women in low socioeconomic status was associated with better childhood nutrition [53,63]. Enabling resources, thus, tend to make a difference in women who belong to a higher wealth status, which could explain why the disparity between women and their husbands is larger in those of higher socioeconomic status compared to those of lower socioeconomic status. This suggests a need to empower women in households with low household wealth to lead to extensive coverage of complete vaccination of children.

Generally, the enhancement of women’s empowerment is indispensable to improve the vaccination level of their child or children. National immunization campaigns should focus on women under unequal power relations, especially those in poor families. It is crucial to “ensure equal access to and equal treatment of women in health care”, to guarantee that women can make healthcare choices on behalf of themselves and their children, as proposed by the Beijing Declaration and Platform for Action [64]. Additionally, as the Sustainable Development Goals (SDGs) are integrated and affect each other, collaborative actions can be taken to achieve SDG#3 on children’s good health through immunization and well-being and SDG#5 on gender equality [65].

The most important strength is that this study is the first to explore the association between women’s empowerment and complete children’s vaccination using MICS data. The MICS is a standardized measurement tool that enables national comparisons. It also provides a sufficiently large and nationally representative sample of women and children in the DRC with population weight in our study. Additionally, it is the first study to investigate the association in the DRC in order to fill the gap in the published literature on this topic in Central Africa. Moreover, instead of piling up the relevant variables, we gave insights into the theme from a comprehensive and systematic viewpoint with a framework and adopted the PCA method to analyze the components of women’s empowerment.

This study, however, had several limitations. Firstly, as this study utilized cross-sectional survey data from MICS, the causal inference that empowered women may ensure complete vaccination of their children could not be drawn; hence, further analysis using cohort data is needed. Secondly, as the survey did not aim to specifically measure women’s empowerment, individual economic empowerment was not well-represented in MICS-6 by direct indicators, which were replaced by variables such as household wealth status and women’s possession of a mobile phone. Finally, the study also failed to examine children who had delayed vaccinations, which is known to increase the likelihood of infection [66] and caused random misclassification in the present study. As a household-based analysis, potential bias may have arisen from migrant populations and missing data; however, we think these influences were minimal. Future research should focus on incorporating more aspects of women’s empowerment to better measure the notion and examine this association in a longitudinal study or a multinational study, and finally to facilitate the implementation of corresponding interventions and policies.

## 5. Conclusions

Using the PCA method, women’s empowerment was extracted into three dimensions, namely intrinsic agency, enabling resources, and social independence. Higher levels of women’s empowerment, especially in terms of intrinsic agency and enabling resources, were associated with better possibility of complete vaccination in children in the DRC, although social independence showed no significant association with the vaccination statuses of children. Household wealth status influenced the associations between empowerment of women and complete vaccination of children, while intrinsic agency had an effect on the women from relatively poor households. Among the richer households, enabling resources affected the vaccination status. As such, the indispensable role of women’s empowerment must be considered in expanding the coverage of complete vaccination in children and promoting equal access to vaccines.

## Figures and Tables

**Table 1:** Principal component analysis rotated factor loadings (N = 12,784).

Items	Intrinsic Agency	Enabling Resources	Social Independence
Considering beating not justified if wife goes out without telling husband	0.793	0.014	0.014
Considering beating not justified if wife neglects the children	0.806	−0.023	0.004
Considering beating not justified if wife argues with husband	0.801	0.053	0.029
Considering beating not justified if wife refuses to have sex with husband	0.755	0.101	0.026
Considering beating not justified if wife burns the food	0.696	0.060	0.011
Women’s highest education level	0.090	0.627	0.017
Covered by health insurance	0.044	0.444	0.066
Owning a mobile phone	0.090	0.653	0.024
Had used the internet	0.020	0.729	0.093
Had used a computer	0.014	0.684	0.084
Age difference: women’s age minus husband’s age	0.005	−0.166	0.301
Women’s age at first marriage	0.035	0.085	0.907
Women’s age at first birthing	0.003	0.018	0.917

**Table 2:** Women’s empowerment-related variables in the DRC according to vaccination status of their children aged 12–23 months (N = 3524) ^1^.

Variables	Total	Complete Vaccination	Incomplete Vaccination	*p*-Value ^2,3^
	100.0	36.6	63.4	
Intrinsic agency, %				<0.001
Low	26.3	27.7	72.3	
Middle	33.6	35.3	64.7	
High	40.1	43.5	56.5	
Enabling resources, %				<0.001
Low	27.1	31.1	68.9	
Middle	28.1	27.6	72.4	
High	44.8	45.5	54.5	
Social independence, %				0.047
Low	33.9	36.4	63.6	
Middle	32.2	33.0	67.0	
High	34.0	40.1	59.9	
Considering beating not justified if wife goes out without telling husband, %				<0.001
No	40.7	30.9	69.1	
Yes	59.3	40.5	59.5	
Considering beating not justified if wife neglects the children, %				<0.001
No	44.1	30.4	69.6	
Yes	55.9	41.5	58.5	
Considering beating not justified if wife argues with husband, %				<0.001
No	46.2	31.8	68.2	
Yes	53.8	40.7	59.3	
Considering beating not justified if wife refuses to have sex with husband, %				<0.001
No	44.0	31.4	68.6	
Yes	56.0	40.6	59.4	
Considering beating not justified if wife burns the food, %				<0.001
No	24.6	25.5	74.5	
Yes	75.4	40.2	59.8	
Woman’s highest education level, %				<0.001
Pre-primary or none	17.5	32.2	67.8	
Primary	34.3	31.8	68.2	
Secondary 1	14.0	30.3	69.7	
Secondary 2	30.1	44.1	55.9	
Higher	4.1	61.4	38.6	
Covered by health insurance, %				<0.001
No	96.2	35.4	64.6	
Yes	3.8	65.8	34.2	
Owning a mobile phone, %				<0.001
No	71.2	29.6	70.4	
Yes	28.8	53.8	46.2	
Had used the internet, %				<0.001
No	95.6	35.4	64.6	
Yes	4.4	62.3	37.7	
Had used a computer, %				<0.001
No	94.8	35.1	64.9	
Yes	5.2	63.2	36.8	
Age difference: woman’s age minus husband’s age, mean (SD)	-6.7 (5.4)	-7.1 (5.1)	-6.5 (5.5)	0.15
Woman’s age at first marriage, mean (SD)	19.0 (4.6)	19.3 (4.8)	18.8 (4.5)	0.002
Woman’s age at first birthing, mean (SD)	20.7 (4.3)	21.1 (4.5)	20.5 (4.2)	0.006

^1^ Weighted using MICS sampling weight. ^2^ Wald Chi-square tests for categorical variables and Student’s *t*-tests for continuous variables. ^3^ Here, *p*-values less than 0.05 considered statistically significant.

**Table 3:** Association between women’s empowerment and children’s complete vaccination in the DRC (N = 3524) ^1^.

Variables	Odds Ratio	95% CI	*p*-Value ^2^
Intrinsic agency			
Low	Ref		
Middle	1.19	(0.86, 1.62)	0.29
High	1.63	(1.19, 2.24)	0.002
Enabling resources			
Low	Ref		
Middle	1.24	(0.88, 1.77)	0.22
High	1.59	(1.11, 2.28)	0.012
Social independence			
Low	Ref		
Middle	0.75	(0.56, 1.02)	0.07
High	1.01	(0.72, 1.40)	0.97
Women’s age	1.01	(0.99, 1.03)	0.36
Children’s sex			
Female	Ref		
Male	0.92	(0.72, 1.18)	0.53
Residence			
Rural	Ref		
Urban	0.66	(0.46, 0.95)	0.027
Family wealth index			
Poor	Ref		
Middle	1.50	(1.07, 2.09)	0.018
Rich	3.06	(1.96, 4.78)	<0.001
Number of household members			
1–5	Ref		
6–10	0.88	(0.68, 1.15)	0.36
≥10	0.67	(0.36, 1.25)	0.20
Number of children aged < 5			
1	Ref		
2	0.71	(0.53, 0.94)	0.015
≥3	0.66	(0.45, 0.98)	0.039
Province			
Sankuru	Ref		
Kinshasa	9.44	(2.42, 36.76)	0.001
Kongo Central	21.84	(5.52, 86.40)	<0.001
Kwango	8.51	(2.19, 33.07)	0.002
Kwilu	3.57	(0.85, 14.97)	0.08
Maindombe	4.53	(1.08, 19.01)	0.039
Équateur	10.99	(2.88, 41.97)	<0.001
Sud Ubangi	13.34	(3.61, 49.27)	<0.001
Nord Ubangi	7.51	(1.86, 30.36)	0.005
Mongala	2.71	(0.52, 14.11)	0.24
Tshuapa	6.72	(1.74, 25.98)	0.006
Tshopo	6.73	(1.74, 25.97)	0.006
Bas Uele	10.60	(2.60, 43.31)	0.001
Haut Uele	7.14	(1.60, 31.96)	0.01
Ituri	21.56	(5.55, 83.75)	<0.001
Nord Kivu	74.83	(18.43, 303.75)	<0.001
Sud Kivu	21.91	(5.91, 81.32)	<0.001
Maniema	2.39	(0.59, 9.72)	0.22
Haut Katanga	16.62	(4.44, 62.18)	<0.001
Lualaba	2.62	(0.64, 10.68)	0.18
Haut Lomami	15.76	(4.27, 58.19)	<0.001
Tanganyika	6.31	(1.56, 25.47)	0.01
Lomami	15.18	(4.05, 56.90)	<0.001
Kasaï Oriental	12.14	(3.20, 46.12)	<0.001
Kasaï Central	22.07	(5.90, 82.53)	<0.001
Kasaï	4.80	(1.27, 18.15)	0.021

^1^ Weighted using MICS sampling weight. ^2^ Here, *p*-values less than 0.05 considered statistically significant.

**Table 4:** Association between women’s empowerment and children’s complete vaccination stratified by wealth status in the DRC ^1,2^.

Variables	Poor (N = 2082)			Middle (N = 684)			Rich (N = 724)		
	Odds Ratio	95% CI	*p*-Value ^3^	Odds Ratio	95% CI	*p*-Value ^3^	Odds Ratio	95% CI	*p*-Value ^3^
Intrinsic agency									
Low	Ref			Ref			Ref		
Middle	1.72	(1.09, 2.70)	0.019	1.73	(0.92, 3.25)	0.09	0.93	(0.50, 1.71)	0.81
High	1.92	(1.23, 2.98)	0.004	2.35	(1.14, 4.85)	0.021	1.65	(0.86, 3.14)	0.13
Enabling resources									
Low	Ref			Ref			Ref		
Middle	1.23	(0.81, 1.88)	0.33	2.19	(1.10, 4.36)	0.026	0.95	(0.30, 3.00)	0.93
High	1.39	(0.83, 2.31)	0.21	1.21	(0.58, 2.50)	0.61	2.62	(1.01, 6.78)	0.047
Social independence									
Low	Ref			Ref			Ref		
Middle	1.19	(0.78, 1.81)	0.42	0.82	(0.42, 1.60)	0.57	0.47	(0.26, 0.83)	0.010
High	1.15	(0.71, 1.87)	0.57	0.90	(0.47, 1.73)	0.75	1.03	(0.55, 1.91)	0.93

^1^ Adjusted for women’s age, children’s sex, residence, number of household members, number of children aged under 5, and province. ^2^ Weighted using MICS sampling weight. ^3^ Here, *p*-values less than 0.05 considered statistically significant.

## Data Availability

Publicly available datasets were analyzed in this study and the data can be found here: http://mics.unicef.org/ (accessed on 11 November 2020).

## Supplementary Materials

The following are available online at https://www.mdpi.com/article/10.3390/vaccines9101117/s1, Table S1. Items of women’s empowerment used in the PCA in the DRC, Table S2. PCA rotated factor loadings for four factors, Table S3. Principal components of women’s empowerment, Table S4. Demographic variables of women and their children in DRC by vaccination status of children aged 12–23 months (N = 3524) ^1^, Table S5. Weighted vaccination coverage of children aged 12–23 months in the DRC in the study sample (N = 3524) ^1^, Figure S1. Scree plot of the PCA analysis.
