# Supplementary material for: Women’s Empowerment and Children’s Complete Vaccination in the Democratic Republic of the Congo: A Cross-Sectional Analysis
Source: Vaccines (Basel). 2021 Oct 1;9(10):1117. doi: 10.3390/vaccines9101117 (PMC8540931; doi:10.3390/vaccines9101117)
Supplement: Supplementary file 1 [file vaccines-09-01117-s001.zip › vaccines-1351594-supplementary.pdf]

Supplementary Materials

Table S1. Items of women's empowerment used in the PCA in the DRC.

Items	Code or Unit
Considering beating not justified if wife goes out without telling husband	Justified or don't know = 0; not justified = 1
Considering beating not justified if wife neglects the children	Justified or don't know = 0; not justified = 1
Considering beating not justified if wife argues with husband	Justified or don't know = 0; not justified = 1
Considering beating not justified if wife refuses to have sex with husband	Justified or don't know = 0; not justified = 1
Considering beating not justified if wife burns the food	Justified or don't know = 0; not justified = 1
Woman's highest education level	Pre-primary/none = 1; Primary = 2; Secondary 1 = 3; Secondary 2 = 4; Higher = 5
Covered by health insurance	Yes = 1; No = 0
Owning a mobile phone	Yes = 1; No = 0
Had used the internet	Yes = 1; No = 0
Had used a computer	Yes = 1; No = 0
Age difference: woman's age minus husband's age	Years
Woman's age at first marriage	Years
Woman's age at first birthing	Years

Table S2. PCA rotated factor loadings for four factors.

Items	Factor 1	Factor 2	Factor 3	Factor 4
Considering beating not justified if wife goes out without telling husband	0.795	0.010	0.037	−0.005
Considering beating not justified if wife neglects the children	0.810	−0.004	0.033	−0.055
Considering beating not justified if wife argues with husband	0.799	0.031	0.015	0.074
Considering beating not justified if wife refuses to have sex with husband	0.751	0.031	0.023	0.134
Considering beating not justified if wife burns the food	0.694	0.013	0.020	0.077
Women's highest education level	0.065	0.055	0.165	0.736
Covered by health insurance	0.030	0.087	0.167	0.469
Owning a mobile phone	0.064	0.062	0.184	0.754
Had used the internet	0.032	0.051	0.848	0.168
Had used a computer	0.030	0.035	0.873	0.076
Age difference: women's age minus husband's age	0.021	0.271	0.115	−0.361
Women's age at first marriage	0.031	0.913	0.047	0.074
Women's age at first birthing	0.002	0.918	0.027	−0.003

Table S3. Principal components of women's empowerment.

Factors	Eigenvalue	Difference	Proportion (%)	Cumulative Proportion (%)
Factor 1	3.113	1.035	23.9	23.9

Factor 2	2.077	0.428	16.0	39.9
Factor 3	1.649	0.611	12.7	52.6
Factor 4	1.039	0.096	8.0	60.6
Factor 5	0.942	0.068	7.3	67.9
Factor 6	0.875	0.245	6.7	74.6
Factor 7	0.629	0.045	4.8	79.4
Factor 8	0.584	0.021	4.5	83.9
Factor 9	0.563	0.108	4.3	88.2
Factor 10	0.455	0.031	3.5	91.7
Factor 11	0.424	0.046	3.3	95.0
Factor 12	0.378	0.107	2.9	97.9
Factor 13	0.271		2.1	100.0

Table S4. Demographic variables of women and their children in DRC by vaccination status of children aged 12–23 months (N = 3524) [1.]

Variables	Total	Complete Vaccination	Incomplete Vaccination	*p*-Value [2,3]
Women's age, mean (SD)	29.6(6.9)	29.8(6.7)	29.5(6.9)	0.32
Child's sex, %				0.83
Female	49.9	36.7	63.3	
Male	50.1	36.5	63.5	
Residence, %				<0.001
Rural	60.6	30.9	69.1	
Urban	39.4	45.3	54.7	
Family wealth index, %				<0.001
Poor	45.3	25.2	74.8	
Middle	19.6	36.4	63.6	
Rich	35.1	51.4	48.6	
Number of household members, %				0.006
1–5	43.6	37.8	62.2	
6–10	50.6	35.8	64.2	
≥10	5.8	34.2	65.8	
Number of children aged < 5, %				0.76
1	31.9	39.7	60.3	
2	52.1	35.6	64.5	
≥3	16.0	33.8	66.3	
Province, %				<0.001
Sankuru	1.3	2.9	97.1	
Kinshasa	10.1	42.7	57.3	
Kongo Central	4.1	55.9	44.2	
Kwango	3.2	24.9	75.1	
Kwilu	5.6	12.8	87.2	
Maindombe	4.1	15.6	84.4	
Équateur	2.3	32.6	67.4	
Sud Ubangi	2.1	28.4	71.6	
Nord Ubangi	0.8	18.6	81.4	
Mongala	0.7	9.2	90.8	
Tshuapa	0.8	16.5	83.5	
Tshopo	3.7	21.9	78.1	
Bas Uele	1.2	23.1	76.9	

Haut Uele	1.6	21.2	78.8
Ituri	4.5	46.9	53.1
Nord Kivu	7.6	78.6	21.4
Sud Kivu	9.3	52.2	47.8
Maniema	1.4	6.5	93.5
Haut Katanga	9.4	48.0	52.0
Lualaba	3.0	12.1	87.9
Haut Lomami	2.2	36.0	64.0
Tanganyika	2.9	21.6	78.4
Lomami	3.9	34.0	66.0
Kasaï Oriental	4.5	33.9	66.1
Kasaï Central	3.9	41.6	58.4
Kasaï	5.8	14.7	85.3

[1] Weighted using MICS sampling weight. [2] Wald Chi-square tests for categorical variables and Student's t-tests for continuous variables. [3] Here, *p*-values less than 0.05 considered statistically significant.

Table S5. Weighted vaccination coverage of children aged 12–23 months in the DRC in the study sample (N = 3524) [1].

Vaccines.	%	95%CI
BCG [2]	73.2	(71.7, 74.6)
Polio [3]	69.8	(68.3, 71.3)
Pentavalent [4]	47.3	(45.7, 49.0)
PCV [5]	45.8	(44.2, 47.4)
Measles [6]	56.9	(55.3, 58.5)
Yellow Fever [7]	55.7	(54.1, 57.4)
Complete vaccination [8]	36.6	(35.0, 38.2)

[1] Weighted using MICS sampling weight. [2] Percentage of children aged 12–23 months who received Bacilli Calmette–Guerin (BCG) vaccine any time prior to the survey. [3] Percentage of children aged 12–23 months who received three doses of polio vaccines (excluding one dose of polio vaccine at birth) at any time before the survey. [4] Percentage of children aged 12–23 months who received three doses of the pentavalent vaccines containing antigens against diphtheria, tetanus, pertussis, hepatitis B, and Hemophilus influenza type b (Hib) at any time before the survey. [5] Percentage of children aged 12–23 months who received three doses of pneumococcal conjugate vaccine (PCV) at any time before the survey. [6] Percentage of children aged 12–23 months who received the measles vaccine at any time before the survey. [7] Percentage of children aged 12–23 months who received the yellow fever vaccine at any time before the survey. [8] Percentage of children aged 12–23 months who received all vaccines recommended below in the National Expanded Program on Immunization (EPI) in the DRC at any time before the survey.

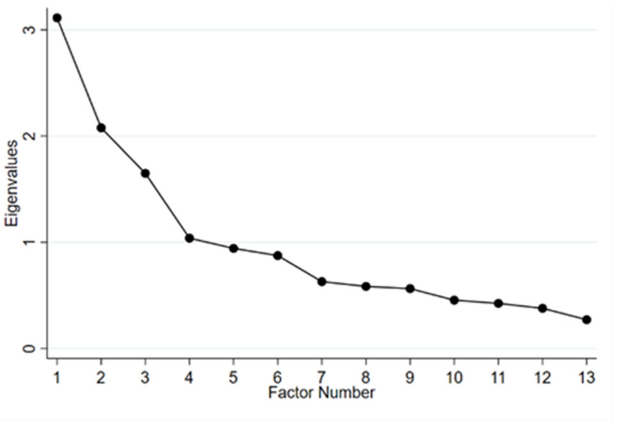

Figure S1. Scree plot of the PCA analysis.
